# Influence of Electric Frequency-to-Place Mismatches on the Early Speech Recognition Outcomes for Electric–Acoustic Stimulation Users

**DOI:** 10.1044/2022_AJA-21-00254

**Published:** 2023-02-17

**Authors:** Margaret T. Dillon, Michael W. Canfarotta, Emily Buss, Meredith A. Rooth, Margaret E. Richter, Andrea B. Overton, Noelle E. Roth, Sarah M. Dillon, Jenna H. Raymond, Allison Young, Adrienne C. Pearson, Amanda G. Davis, Matthew M. Dedmon, Kevin D. Brown, Brendan P. O'Connell

**Affiliations:** a Department of Otolaryngology/Head & Neck Surgery, School of Medicine, The University of North Carolina at Chapel Hill; b Division of Speech and Hearing Sciences, Department of Allied Health Sciences, The University of North Carolina at Chapel Hill; c Department of Audiology, UNC Health, Chapel Hill, NC

## Abstract

**Purpose:**

Cochlear implant (CI) recipients with hearing preservation experience significant improvements in speech recognition with electric–acoustic stimulation (EAS) as compared to with a CI alone, although outcomes across EAS users vary. The individual differences in performance may be due in part to default mapping procedures, which result in electric frequency-to-place mismatches for the majority of EAS users. This study assessed the influence of electric mismatches on the early speech recognition for EAS users.

**Method:**

Twenty-one participants were randomized at EAS activation to listen exclusively with a default or place-based map. For both groups, the unaided thresholds determined the acoustic cutoff frequency (i.e., > 65 dB HL). For default maps, the electric filter frequencies were assigned to avoid spectral gaps in frequency information but created varying magnitudes of mismatches. For place-based maps, the electric filter frequencies were assigned to avoid frequency-to-place mismatches. Recognition of consonant–nucleus–consonant words and vowels was assessed at activation and 1, 3, and 6 months postactivation.

**Results:**

For participants with default maps, electric mismatch at 1500 Hz ranged from 2 to −12.0 semitones (*Mdn* = −5 semitones). Poorer performance was observed for those with larger magnitudes of electric mismatch. This effect was observed through 6 months of EAS listening experience.

**Conclusions:**

The present sample of EAS users experienced better initial performance when electric mismatches were small or eliminated. These data suggest the utility of methods that reduce electric mismatches, such as place-based mapping procedures. Investigation is ongoing to determine whether these differences persist with long-term EAS use.

**Supplemental Material:**

https://doi.org/10.23641/asha.22096523

Adult cochlear implant (CI) recipients with hearing preservation experience significant improvements in speech recognition with electric–acoustic stimulation (EAS) as compared to preoperative performance or postactivation with a CI alone, although performance across EAS users remains widely variable (Dillon et al., 2015; Gantz et al., 2016; Gstoettner et al., 2008; Kiefer et al., 2005; Pillsbury et al., 2018). This variability may be due in part to individual differences in residual hearing and angular insertion depth (AID) of the electrode contacts, resulting in electric frequency-to-place mismatches. Electric frequency-to-place mismatches are discrepancies between the electric filter frequencies and the cochlear place frequencies for individual electrode contacts, which introduce spectral shifts in the provided speech information relative to the cochlear tonotopicity.

Maps that align the filter frequencies to the cochlear place frequencies, referred to here as *place-based maps*, have been associated with better speech recognition than spectrally shifted maps based on data from CI-alone vocoder simulations (Başkent & Shannon, 2003; Dorman et al., 1997; Li & Fu, 2010) and in CI-alone device users (Başkent & Shannon, 2004, 2005; Fu & Shannon, 1999). Performance tends to improve after training or prolonged listening experience with spectrally shifted maps for both CI-alone simulations and devices (Faulkner, 2006; Fu & Galvin, 2003; Fu et al., 2002, 2005; Li & Fu, 2007; Li et al., 2009; Reiss et al., 2007; Rosen et al., 1999; Sagi et al., 2010; Smalt et al., 2013; Svirsky et al., 2004, 2015), although performance may not reach the level achieved with a place-based map (Li et al., 2009; Rosen et al., 1999). For EAS users, the impact of electric mismatches on speech recognition is less clear. The low-frequency acoustic hearing could serve as an anchor to cochlear tonotopicity, making it more challenging to acclimate to the spectrally shifted mid- and high-frequency electric information. Alternatively, low-frequency acoustic hearing could facilitate acclimatization by reducing the perceptual distance between speech encoded using EAS and the linguistic templates in memory, formed based on full-bandwidth acoustic hearing. It is unknown if electric mismatches influence the early speech recognition of EAS users or if users can acclimate to spectrally shifted maps within the initial months of listening experience.

Default mapping procedures for EAS devices do not incorporate variability in AID of the electrode contacts, which can result in electric frequency-to-place mismatches that negatively influence acute speech recognition (Dillon, Canfarotta, Buss, Hopfinger, & O'Connell, 2021; Dillon et al., 2022; Fu et al., 2017; Willis et al., 2020). The AID of the electrode contacts varies widely across CI recipients due to differences in electrode array design, surgical approach, and cochlear morphology (Canfarotta et al., 2020; Landsberger et al., 2015). Default mapping procedures for EAS devices are designed to prioritize providing the listener with the full speech spectrum, split between the acoustic and electric components. Unaided detection thresholds in the implanted ear are used to identify the frequency at which thresholds exceed a functional hearing level criterion (e.g., 65 dB HL). This frequency is assigned as both the acoustic high-frequency cutoff and the lowest-frequency filter for electric stimulation from the most apical electrode contact to avoid spectral gaps in the speech information provided to the listener. The majority of EAS users experience spectral shifts of 6 semitones (half octave) or more with default maps (Canfarotta et al., 2020). Recent EAS simulation studies have shown better acute speech recognition with place-based maps than spectrally shifted maps (Dillon, Canfarotta, Buss, Hopfinger, & O'Connell, 2021; Dillon et al., 2022; Fu et al., 2017; Willis et al., 2020). However, it is unclear whether these performance differences would be observed for actual EAS users.

EAS users may experience better early speech recognition with maps derived from a place-based mapping procedure that eliminates electric frequency-to-place mismatches by incorporating the AID of electrode contacts for individual CI recipients. For example, the place-based mapping procedure used in this report calculated the AID of each electrode contact from the postoperative computed tomography (CT) imaging and estimated the cochlear place frequency. The filter frequencies for specific channels were assigned to match the place frequencies of the corresponding electrode contacts. While this approach eliminates electric mismatches, it can result in spectral gaps that default mapping procedures avoid. Spectral gaps generally result in poorer performance for EAS users (Gifford et al., 2017; Karsten et al., 2013), although recent data using EAS simulations suggest that the benefit of place-based mapping may outweigh the detrimental effect of a spectral gap under some conditions (Dillon, Canfarotta, Buss, Hopfinger, & O'Connell, 2021; Fu et al., 2017; Willis et al., 2020).

This report assessed the influence of electric frequency-to-place mismatches on the speech recognition outcomes of EAS users within the first 6 months of device use. The study sample included EAS users who were randomized to listen exclusively with either a default map (variable magnitudes of electric mismatch across individuals) or a place-based map (no electric mismatch). The hypotheses were that early speech recognition outcomes would be better for EAS users with smaller electric mismatches and that the negative influence of larger electric mismatches would persist over the initial months of EAS listening experience.

## Method

Preliminary data were reviewed from an ongoing, prospective investigation of performance with default versus place-based maps for CI recipients. The study site institutional review board approved the procedures, and participants provided consent. Participants were randomized to listen exclusively with either a default or place-based map. Procedures were completed at device activation (2–4 weeks postoperatively) and at 1, 3, and 6 months postactivation. Both the research audiologist who completed the assessments and the participants were blinded to the assigned map.

### Participants

Adult CI recipients were considered for inclusion if they underwent cochlear implantation at the study site, received a MED-EL lateral wall electrode array, were 18–80 years of age at the time of surgery, and presented with an unaided hearing threshold of ≤ 65 dB HL at 125 Hz in the implanted ear at device activation. Potential participants who failed the Mini-Mental State Examination (Folstein et al., 1975) or reported cognitive issues were excluded.

### Procedure

Unaided detection thresholds in the implanted ear were measured behaviorally using pure-tone stimuli presented over insert earphones at each interval. A low-frequency pure-tone average (LFPTA) was calculated from the unaided detection thresholds at 125, 250, and 500 Hz.

Participants were fit with the ear-level Sonnet2^EAS^ processor. The acoustic component was fit based on the participant's unaided detection thresholds in the implanted ear. The thresholds were entered into the clinical programming software (Maestro v9), which determined the acoustic cutoff frequency by identifying the frequency at which the detection threshold exceeded 65 dB HL. The acoustic settings were verified using the NAL-NL1 prescriptive targets (Byrne et al., 2001), using either real-ear or test box measures with the Verifit2 hearing instrument analyzer (Audioscan). A value of 120 dB HL was entered to indicate no response at the output limit of the audiometer. For the CI component, the maximum comfortable loudness (MCL) levels were measured behaviorally for each channel individually and then loudness was balanced across channels using the “adjacent-reference method” (Throckmorton & Collins, 2001; Zwolan et al., 1997). The threshold (T) levels for the map were 10% of the MCL level for each channel at initial activation; at the postactivation intervals, behavioral thresholds were measured, and T levels were set just below detection threshold.

For participants randomized to listen with a default map, the electric filter frequencies were generated by the clinical software. The software assigned the low-frequency filter associated with the most apical electrode contact as the frequency where the unaided detection threshold exceeded 65 dB HL (the acoustic cutoff frequency). The remaining mid- to high-frequency information was logarithmically distributed across the active channels.

For participants randomized to listen with a place-based map, the electric filter frequencies were assigned to align low- to mid-frequency information with the cochlear place frequencies. Postoperative CT imaging was obtained for all cases using a Morita cone-beam CT scanner, and the image was uploaded to the OTOPLAN software (CAScination AG and MED-EL Corporation). Two reviewers manually identified cochlear anatomical landmarks (e.g., modiolus and round window) and individual electrode contacts for each CT image; those landmarks were used by the software to calculate the AID for each electrode contact, as previously described (Canfarotta et al., 2019). The cochlear place frequency for individual electrode contacts was then estimated using a spiral ganglion (SG) frequency-to-place function (Stakhovskaya et al., 2007). The SG function was selected based on a pilot study showing better acute speech recognition with place-based maps using the SG function as compared to an organ of Corti function (Dillon, Canfarotta, Buss, & O'Connell, 2021). The filter frequencies matched the place frequency for electrode contacts residing up to at least the 3-kHz cochlear region. The remaining high-frequency information was logarithmically distributed across the more basal electrode contacts. For electrode contacts at cochlear place frequencies in the region of functional acoustic hearing (i.e., unaided detection threshold ≤ 65 dB HL), the MCL level was set below threshold (e.g., at 1 charge unit [qu]) with T levels at 10% of the MCL level. For example, if a participant had an unaided threshold of 65 dB HL at 500 Hz, the stimulation level for an electrode contact at a cochlear place frequency ≤ 500 Hz would be reduced. The rationale for this procedure was that it may limit potential electric-on-acoustic masking (e.g., Imsiecke, Krüger, et al., 2020).

Speech recognition was assessed using tasks of vowel and word recognition. Vowel recognition tasks have been shown to be sensitive measures of the influence of electric mismatches on speech recognition with a CI alone (Başkent & Shannon, 2003, 2005; Fu et al., 2002). The assessment of vowel recognition was completed in a quiet room with a direct connect setup. The participant's processor was connected to the computer using a 90/10 direct audio input cable. Twelve vowel sounds were presented in an /h/−vowel−/d/ context using the English Vowel Recognition Test from TigerSpeech Technology. Participants listened to the target and selected the perceived vowel from a closed-set list. The assessment of word recognition was completed in a double-walled soundbooth, with the participant seated 1 m from the loudspeaker. Performance was assessed with consonant–nucleus–consonant (CNC) words (Peterson & Lehiste, 1962). The recorded 50-word lists were presented at 60 dB SPL, and the contralateral ear was masked. The tester scored each verbal response as correct or incorrect. Performance was scored as the percent correct for both tasks. The tasks were completed after mapping at the activation interval and before mapping at the postactivation intervals.

### Electric Frequency-to-Place Mismatch

Electric frequency-to-place mismatch was quantified as the semitone deviation between the electric center frequency and the SG place frequency for the electrode contact closest to the 1500-Hz cochlear place (~267°). The 1500-Hz place frequency was selected because it has been shown to be an important region for frequency alignment in CI simulations evaluating speech recognition (Başkent & Shannon, 2007).

### Data Analysis

Linear mixed models (LMMs) implemented in R using the *lme* package (R Studio Team, 2020) assessed the main effects of electric mismatch at 1500 Hz, interval, AID of E1, and the interaction of electric mismatch and interval on speech recognition, with participant included as a random factor. Percent correct data were converted to rationalized arcsine units (RAUs; Studebaker, 1985) to normalize the variance. In the statistical models, electric mismatch was converted to an absolute value, removing the distinction between basal and apical shifts.

## Results

**Figure 1.**
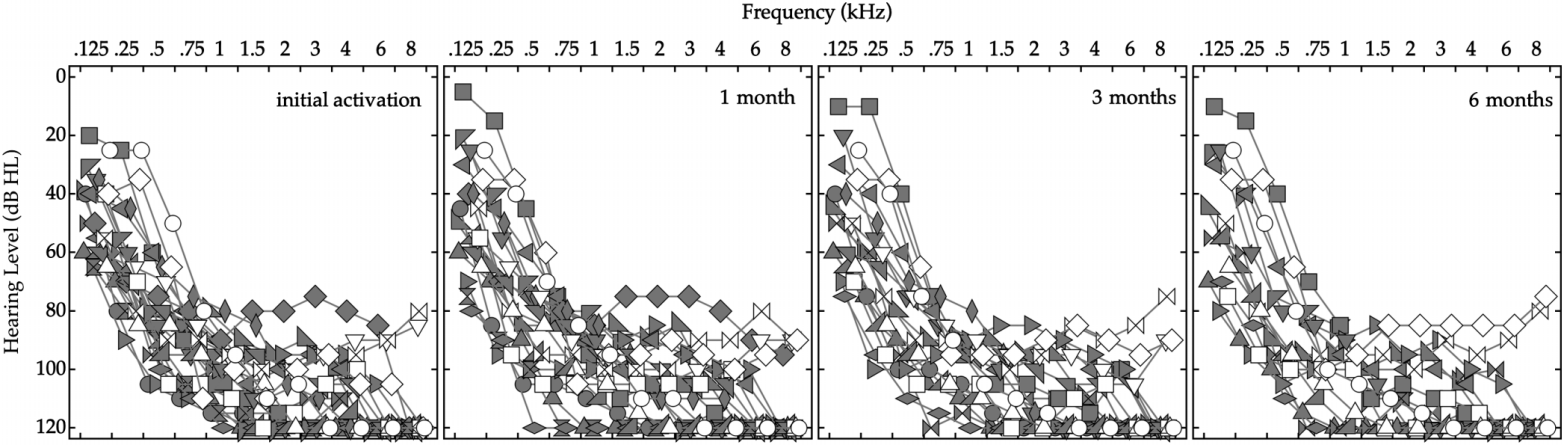
Unaided air-conduction pure-tone detection thresholds for each participant at initial activation and 1, 3, and 6 months postactivation. Individual thresholds are indicated by symbol shape and fill, as defined in Table 1.

The data included 21 participants (11 female) who were randomized to listen with either a default map (*n* = 15) or a place-based map (*n* = 6). Age at implantation ranged from 22 to 78 years, with a median of 66 years. Eleven participants were implanted with a Flex24 array; six, with a Flex28 array; and four, with a FlexSOFT array. All cases had a full insertion of the electrode array. The median AID of E1 was 503°, with a range of 370°–691°. For participants with default maps, electric mismatch at 1500 Hz ranged from 2 to −12.0 semitones (*Mdn* = −5 semitones),^1^ with positive values indicating an apical shift and negative values indicating a basal shift of the spectral information relative to the cochlear place frequency. At the time of data review, there were more participants randomized to default maps than place-based maps, which provided a sufficient spread of electric mismatches to assess the influence on early performance. For participants with place-based maps, two participants (PB1 and PB3) had spectral gaps, and four participants (PB2, PB4, PB5, and PB6) had at least one electrode contact with stimulation levels reduced below threshold. Table 1 lists the demographic information for the sample; participants are ordered by AID of E1. Figure 1 plots the unaided detection thresholds for each participant at the initial activation and at the three postactivation intervals. Symbol shape and fill indicate the thresholds for each participant, as defined in Table 1. Descriptive statistics for speech recognition at each interval are listed in Supplemental Material S1.

**Table 1.** Demographic information for electric–acoustic stimulation users listening with either a default map or a place-based map.

Participant/symbol	Sex	Age (years)	Electrode array	AID of E1		Acoustic cutoff (Hz)	Electrode at 1500 Hz	Electric mismatch (semitone deviation)	Reduced channel(s)
				Degrees	Hz				
D1 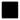	M	66	Flex24	370	809	542	4	−5	
D2 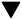	F	74	Flex24	404	671	300	4	−11	
D3 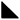	M	78	Flex24	410	650	250	4	−12	
PB1 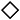	F	45	Flex24	423	606	500	5	0	
PB2 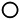	M	50	Flex24	428	590	625	5	0	E1
D4 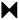	M	78	Flex24	429	587	286	5	−9	
D5 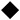	F	69	Flex24	452	520	219	5	−11	
D6 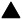	F	66	Flex24	483	442	187	5	−8	
D7 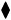	F	52	Flex24	487	433	500	5	−1	
D8 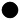	F	54	Flex24	495	415	203	5	−8	
D9 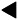	F	53	Flex24	503	398	563	5	2	
D10 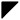	F	73	Flex28	504	396	350	5	−3	
PB3 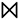	M	73	Flex28	530	345	175	6	0	
D11 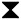	M	67	Flex28	533	339	125	6	−5	
D12 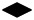	F	57	Flex28	542	323	208	6	−5	
D13 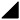	M	69	Flex28	545	318	250	6	−5	
PB4 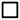	M	52	FlexSOFT	575	269	214	6	0	E1
D14 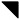	M	69	Flex28	616	211	250	6	−2	
PB5 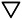	M	54	FlexSOFT	669	144	250	7	0	E1, E2
D15 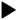	F	22	FlexSOFT	689	120	125	6	−1	
PB6 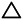	F	67	FlexSOFT	691	118	125	6	0	E1

*Note.* Participants are sorted by the angular insertion depth (AID) of the most apical electrode contact (E1). Symbol fill indicates whether the participant listened with a default (filled) or place-based (open) map. “Age” refers to age at the time of cochlear implantation (years). Acoustic cutoff (Hz) was determined at initial activation.

**Figure 2.**
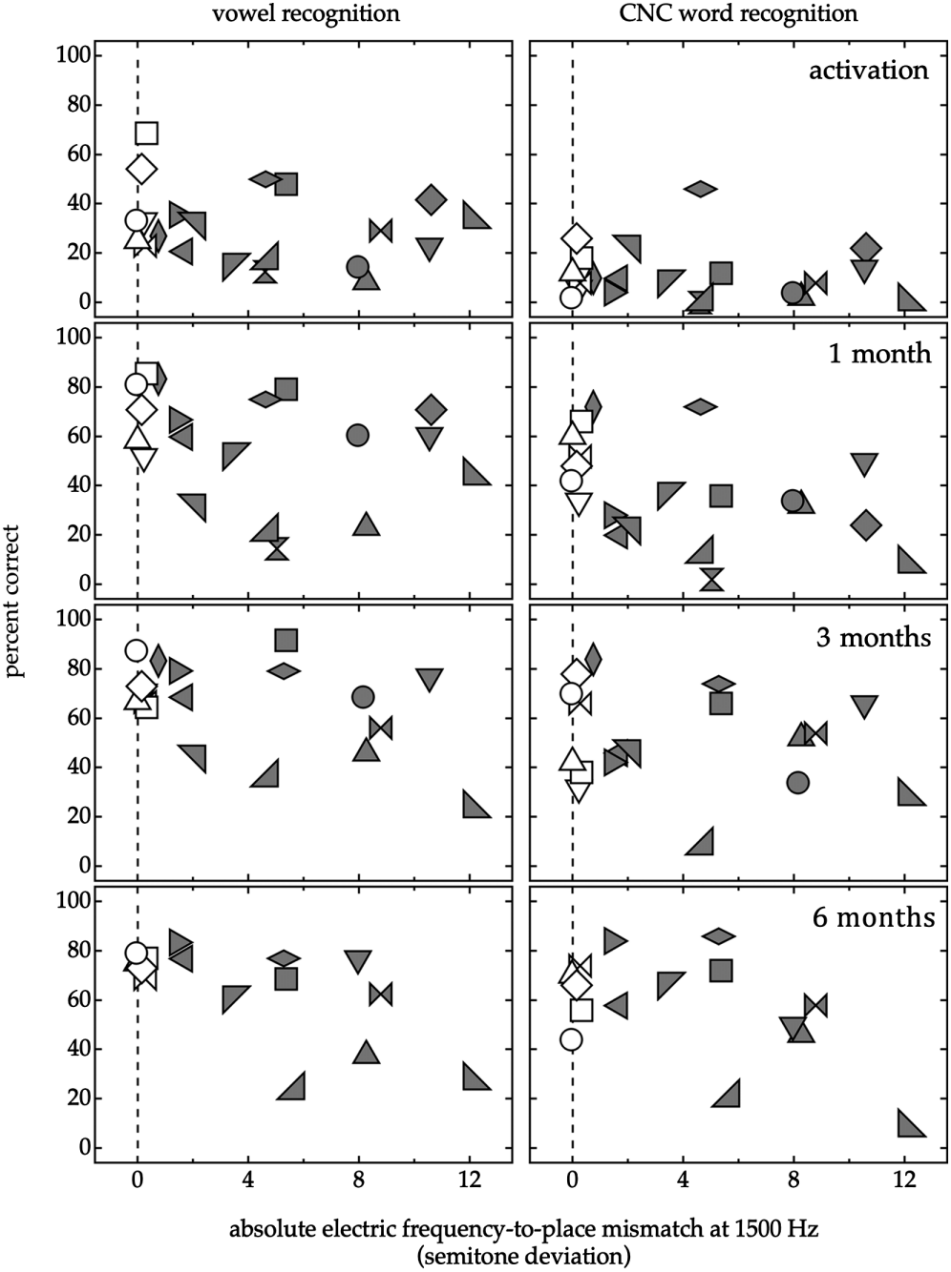
Speech recognition as a function of electric frequency-to-place mismatch at 1500 Hz at initial activation and 1, 3, and 6 months postactivation. Performance was assessed for vowel recognition (left column) and consonant–nucleus–consonant (CNC) words recognition (right column), scored as the percent correct. Individual performance is indicated by symbol shape and fill, as defined in Table 1. The vertical dashed line at 0 semitones indicates the alignment between the channel center frequency and the cochlear place frequency for the electrode contact closest to 1500 Hz.

Figure 2 plots the speech recognition over time on the vowel recognition task (left column) and CNC words test (right column) as a function of electric mismatch at 1500 Hz. The LMMs included data from the postactivation intervals (1, 3, and 6 months); data from the initial activation were not included due to floor performance for some participants. This report aimed to assess the influence of electric mismatches on the speech recognition outcomes of EAS users within the first 6 months of device use. LMMs first assessed the data from participants with default maps since the electric filters for the participants with place-based maps had been adjusted to eliminate mismatches. Table 2 lists the coefficients for both LMMs. The main effect of interval just missed significance for vowel recognition, *F*(2, 16) = 3.6, *p* = .050, and achieved significance for CNC word recognition, *F*(2, 16) = 23.5, *p* < .001, with participants experiencing improvements in speech recognition over time. For CNC words, there was a significant interaction between interval and electric mismatch, *F*(2, 16) = 11.7, *p* < .001, reflecting the fact that participants with minimal mismatches experienced more substantial improvements in performance over time than participants with larger magnitudes of mismatch. This interaction between interval and mismatch was not observed for vowel recognition, *F*(2, 16) = 1.4, *p* = .283.

**Table 2.** Regression coefficients from the linear mixed models that evaluated the main effects of interval (1, 3, and 6 months), absolute electric frequency-to-place mismatch at 1500 Hz, angular insertion depth (AID) of E1, and the interaction of interval and electric mismatch on speech recognition (rationalized arcsine units) for participants with default maps.

	Vowel recognition					CNC words recognition				
	Coefficient	*SE*	*df*	*t* value	*p* value	Coefficient	*SE*	*df*	*t* value	*p* value
3 months	**13.93**	**6.07**	**16**	**2.29**	**.036**	**14.80**	**6.09**	**16**	**2.43**	**.027**
6 months	**15.71**	**7.25**	**16**	**2.17**	**.046**	**49.79**	**7.27**	**16**	**6.85**	**< .001**
Electric mismatch	−2.85	1.76	16	−1.62	.124	−1.55	1.84	16	−0.84	.413
AID	−0.13	0.08	13	−1.60	.133	−0.08	0.09	13	−0.96	.352
3 Months × Electric Mismatch	−0.95	0.92	16	−1.04	.315	−0.04	0.92	16	−0.04	.965
6 Months × Electric Mismatch	−1.77	1.10	16	−1.61	.127	**−4.84**	**1.10**	**16**	**−4.39**	**.001**

*Note.* Significant results are indicated in bold. The 1-month postactivation visit was the reference for interval. E1 = Electrode 1 (most apical electrode); CNC = consonant–nucleus–consonant.

In a second set of analyses, these LMMs were expanded to include the data from the participants with place-based maps. Table 3 lists the coefficients for both LMMs. There was a significant main effect of electric mismatch for vowel recognition, *F*(1, 26) = 5.5, *p* = .027, and for CNC word recognition, *F*(1, 27) = 4.6, *p* = .041, with poorer performance observed for participants with larger magnitudes of electric mismatch on both tasks. The interaction of electric mismatch and interval was not significant for vowel recognition, *F*(2, 26) = 0.59, *p* = .561, or for CNC word recognition, *F*(2, 27) = 1.4, *p* = .252. Taken together, these results indicate that EAS users with smaller magnitudes or no electric mismatch had better speech recognition scores than EAS users with larger magnitudes of electric mismatch over the first 6 months of listening experience.

**Table 3.** Regression coefficients from the linear mixed models that evaluated the main effects of interval (1, 3, and 6 months), absolute electric frequency-to-place mismatch at 1500 Hz, angular insertion depth (AID) of E1, and the interaction of interval and electric mismatch on speech recognition (rationalized arcsine units) for participants with default maps and participants with place-based maps.

	Vowel recognition					CNC words recognition				
	Coefficient	*SE*	*df*	*t* value	*p* value	Coefficient	*SE*	*df*	*t* value	*p* value
3 months	7.53	4.02	26	1.87	.072	8.84	5.83	27	1.52	.141
6 months	8.59	4.45	26	1.93	.065	**21.76**	**6.43**	**27**	**3.39**	**.002**
Electric mismatch	**−2.69**	**1.15**	**26**	**−2.34**	**.027**	**−2.72**	**1.27**	**27**	**−2.14**	**.041**
AID	−0.08	0.05	19	−1.74	.098	−0.06	0.05	19	−1.29	.213
3 Months × Electric Mismatch	−0.15	0.73	26	−0.21	.837	0.91	1.07	27	0.85	.403
6 Months × Electric Mismatch	−0.83	0.81	26	−1.03	.314	−1.06	1.19	27	−0.89	.379

*Note.* Significant results are indicated in bold. The 1-month post-activation visit was the reference for interval. E1 = Electrode 1 (most apical electrode); CNC = consonant–nucleus–consonant.

Additional analyses were conducted to evaluate other factors thought to affect performance in EAS users (i.e., LFPTA and age). No significant main effects were observed when the models that included the data from all participants were expanded to include LFPTA (*p* ≥ .259); age at implantation was not significant for CNC words (*p* = .510) and trended toward significance for vowel recognition (*p* = .056).

## Discussion

This report prospectively evaluated the early speech recognition for EAS users as a function of electric mismatch. Participants listened exclusively with either default maps (median electric mismatch: −5 semitones) or place-based maps that eliminated electric mismatches for low- to mid-frequency information. Poorer speech recognition was observed for EAS users with larger magnitudes of electric mismatch. For example, models including all participants predict poorer performance at 6 months postactivation for individuals listening to maps with 6 semitones of electric mismatch as compared to maps with no electric mismatch by 21 and 22 RAUs for vowel and word recognition, respectively. These data suggest that electric frequency-to-place mismatches may influence the speech recognition outcomes of adult EAS users—at least within the initial 6 months of device use. The preliminary patterns of performance for EAS users with place-based maps suggest the utility of methods that minimize or eliminate electric mismatches to support early speech recognition.

The early effects of electric frequency-to-place mismatches for EAS users corroborate the previously observed performance differences for participants with normal hearing listening to EAS simulations. In both paradigms, we observe better performance with place-based maps than for spectrally shifted maps (Dillon, Canfarotta, Buss, Hopfinger, & O'Connell, 2021; Dillon et al., 2022; Fu et al., 2017; Willis et al., 2020). For instance, Fu et al. (2017) observed better performance with simulations of a place-based map and a spectrally shifted map with minimal mismatches as compared to spectrally shifted maps with larger magnitudes of mismatch. In this report, EAS users experienced better performance when electric mismatches were small, either with default maps that created minimal spectral shifts or with place-based maps. These findings are compelling considering the limited range of electric mismatches in the present dataset (2 to −12 semitones). EAS users with similar low-frequency acoustic hearing and who received shorter arrays, or a partial insertion with comparable length arrays, could experience even larger electric mismatches when listening with default maps.

One factor to consider when evaluating the present place-based mapping procedure is that it may result in a spectral gap between the acoustic and electric outputs. Default mapping procedures limit spectral gaps by assigning a single frequency to the acoustic cutoff and electric low-frequency filter. The poorer performance with spectral gaps for EAS users and listeners for EAS simulations (Dorman et al., 2005; Gifford et al., 2017; Karsten et al., 2013) has not been observed for simulations of place-based maps (Dillon, Canfarotta, Buss, Hopfinger, & O'Connell, 2021; Fu et al., 2017; Willis et al., 2020). The present sample included two EAS users with place-based maps that created a spectral gap (PB1 and PB3), although the sizes of those gaps were minimal (PB1: 500–508 Hz; PB3: 175–189 Hz). Data for EAS users with placed-based maps and larger spectral gaps are needed to determine the size and frequency range for which a gap may occur before performance is negatively impacted by place-based mapping.

Another factor to consider is electric-on-acoustic masking. For CI recipients with electrode contacts that reside close to or within the region of functional acoustic hearing, the electric current spread from apical electrode contact(s) may introduce substantial masking of the low-frequency acoustic cues (Imsiecke, Büchner, et al., 2020; Imsiecke, Krüger, et al., 2020; Kipping et al., 2020; Koka & Litvak, 2017; Krüger et al., 2017, 2020a, 2020b; Lin et al., 2011). This consideration is increasingly relevant as hearing preservation has been shown in CI recipients of long (e.g., 31.5 mm), flexible lateral wall arrays (Hollis et al., 2021; Mick et al., 2014; Usami et al., 2014). The place-based mapping procedure used in this study aimed to limit potential electric-on-acoustic masking by reducing the stimulation levels for electrode contacts at AIDs that were within the region of functional acoustic hearing. While clinically feasible, this method does not take into consideration spread of excitation from electrode contacts basal to the region of acoustic hearing. Future investigation is needed to determine whether performance with place-based maps would improve using other techniques for avoiding electric-on-acoustic masking (see Imsiecke, Krüger, et al., 2020).

These preliminary data indicate that electric frequency-to-place mismatches influence the early performance of EAS users, although there are limitations worth consideration. This report estimated the cochlear place frequency using the SG frequency-to-place function described by Stakhovskaya et al. (2007). This function was selected based on our previous data showing significantly better performance at device activation for CI alone and EAS users with place-based maps using the SG function as compared to an organ of Corti function (Dillon, Canfarotta, Buss, & O'Connell, 2021). Both frequency-to-place functions make some assumptions about the individual's cochlear morphology. Additional investigation is needed to optimize selection of models for place-based mapping, including consideration of individualized tonotopic models (see Helpard et al., 2021).

Another limitation is the modest sample size of the current study, which is not sufficient to assess the relationship between electric mismatches, AID of E1, and other device and mapping variables that have been observed to influence the speech recognition of CI recipients, such as angular separation between the electrode contacts (Canfarotta et al., 2020; Zhou, 2017) and filter bandwidth (Fu & Shannon, 2002). Participant recruitment is ongoing; a larger dataset will also allow us to consider the differential effects of these factors, as well as possible differences between positive and negative shifts and the influence of electric frequency-to-place mismatches on the binaural hearing for EAS users.

## Conclusions

These preliminary data suggest that EAS users experience better speech recognition when electric frequency-to-place mismatches are minimal and that the negative effects of larger magnitudes of electric mismatches are observed up to 6 months of listening experience. Methods to minimize electric mismatches, such as place-based mapping procedures, may support better early speech recognition for the population of patients similar to the participants in the present experiment. Ongoing work will evaluate the patterns of speech recognition and acclimatization to varying magnitudes of electric frequency-to-place mismatches with long-term device use.

## Data Availability Statement

The present preliminary data are available by e-mailing Margaret T. Dillon (mdillon@med.unc.edu).

## Supplementary Material

10.1044/2022_AJA-21-00254SMS1 Supplemental Material S1 Descriptive statistics of the vowel recognition and consonant–nucleus–consonant (CNC) word recognition (percent correct) for participants with default maps and participants with place-based maps at each interval.

## Footnote

1 Twelve semitones equal one octave. In the statistical models, electric mismatch was converted to an absolute value, removing the distinction between basal and apical shifts.
