# Supplementary material for: Influence of Electric Frequency-to-Place Mismatches on the Early Speech Recognition Outcomes for Electric–Acoustic Stimulation Users
Source: Am J Audiol. 2023 Feb 17;32(1):251–60. doi: 10.1044/2022_AJA-21-00254 (PMC10166189; doi:10.1044/2022_AJA-21-00254)
Supplement: Supplemental Material S1 [file AJA-32-251-s001.pdf]

Supplemental Material S1. Descriptive statistics of the vowel recognition and consonant–nucleus–consonant (CNC) word recognition (percent correct) for participants with default maps and participants with place-based maps at each interval.

		**Vowel Recognition**		**CNC Words Recognition**	
		**Default**	**Place-Based**	**Default**	**Place-Based**
Initial activation	min	8	23	0	2
	median	*27*	*33*	*10*	*10*
	max	50	69	46	26
1 month	min	15	52	2	34
	median	*60*	*71*	*30*	*50*
	max	83	85	72	66
3 months	min	23	65	8	32
	median	*69*	*69*	*50*	*54*
	max	92	88	84	78
6 months	min	23	69	8	44
	median	*66*	*75*	*58*	*66*
	max	83	79	86	74
